# Malaria parasitaemia and its impact on biological parameters among children <16 years old attending the Nkwen District Hospital, Cameroon

**DOI:** 10.5281/zenodo.10731943

**Published:** 2024-03-01

**Authors:** Bertrand Njewa, Ebanga Echi Joan Eyong, Calvin Bissong Ebai

**Affiliations:** 1 Department of Microbiology and Parasitology, Faculty of Science, The University of Bamenda, P.O. Box 39, Bambili, North West Region, Cameroon.; 2 Department of Medical Laboratory Science, Faculty of Health Sciences, The University of Bamenda, P.O. Box 39, Bambili, North West Region, Cameroon.

## Abstract

**Introduction:**

Malaria remains a major public health problem in children in endemic areas. This study aimed to determine its prevalence, intensity, and assess how biological parameters like RBC count, haemoglobin, haematocrit, glycaemia, platelet count and WBC count vary with respect to parasitaemia in children <16 years attending the Nkwen District Hospital, northwest Cameroon.

**Materials and Methods:**

The study was a hospital-based cross-sectional study conducted between March-May 2023. Structured, closed-ended questionnaires were administered to obtain information. Patients’ temperature was measured using an infrared forehead digital thermometer. Malaria was diagnosed by RDT and positive samples Giemsa-stained for parasitaemia. Full blood count was performed using a haemolyser and glycaemia measured using a glucometer.

**Results:**

In total, 321 children were examined. Overall prevalence of malaria (all *P. falciparum*) was 22.7% (73/321), with 24.7% (18/73), 34.2% (25/73) and 41.1% (30/73) having low, moderate and high parasitaemias, respectively. Overall GMPD was 2.670.8±179.9/μL; children aged 6-10 years were hit hardest (5.377.7 ± 3.2/μL). Malaria-positive children had significantly lower RBC count, Hb concentration, Hct, blood sugar, WBC and platelet counts (p<0.05) compared to those that were negative. Among positive children, RBC count, Hct, Hb, lymphocyte and platelet count each showed a significant (p<0.05) decrease while total WBC and granulocyte count each showed a significant (p<0.05) increase with increasing levels of parasitaemia.

**Conclusions:**

Changes in biological parameters during malaria are sensitive but poor specific indicators of malaria because they may overlap with symptoms of other infections. More attention should be given to children aged 6-10 years during strategic planning and design of malaria control programmes.

## Introduction

Malaria is a parasitic disease of humans caused by a protozoan of the genus *Plasmodium,* which is transmitted by a female *Anopheles* mosquito. It has a worldwide distribution but is most prevalent in tropical and subtropical countries [1,2]. Today, approximately 3.3 billion people in 97 countries are at risk of contracting malaria, leading to an estimated 240 million cases and about 600,000 deaths annually [3].

The introduction of malaria parasites into the host peripheral blood by an infected female *Anopheles* mosquito triggers changes in several host biological parameters such as body temperature, haemoglobin concentration, blood sugar level, blood pressure etc., many of which play an important role in malaria pathogenesis [4]. Apart from haemoglobin concentration, other haematological parameters that are often affected include the relative numbers of circulating cell types such as erythrocytes, platelets, granulocytes and lymphocytes These changes may subsequently affect the general physiology of the host, resulting in several clinical manifestations with fever, anaemia and thrombocytope-nia being the most common [4].

In malaria infection, anaemia is associated with significant morbidity and mortality, with higher rates among children particularly in sub-Saharan Africa [5]. Many studies have proven the association of lower haemoglobin concentration with malaria parasitaemia in young children making anaemia an inevitable outcome of malaria infection [6-8]. In a study carried out by Bushra *et al.* [9], there were significant decreases in the values of haemoglobin among all participants infected with *P. falciparum* as compared to the healthy subjects. Another indicator of anaemia, haematocrit (HCT) has also been implicated in malaria infections. Lemma *et al.* [10], observed a 45% decrease in HCT levels among malaria-infected patients. According to several studies, low platelet count (thrombocy-topenia) is the most common haematological abnormality in patients with acute malaria. In their investigation, Kochar *et al.* [11] found that 24.6% of malaria patients had thrombocytopenia, making the relationship between malaria and thrombocy-topenia evident, although its specific pathogenic mechanism remains unclear. While erythrocyte and platelet levels are consistently decreased in malaria-infected individuals, there have been conflicting reports on the effect of malaria on leukocyte counts [4]. A recent study showed a significant reduction in leukocyte count (leukocytopenia is also referred to as leukopenia) in malaria-infected study participants compared with their uninfected counterparts [4]. However, another study reported elevated leukocyte levels (leucocytosis) in malaria-infected study participants compared to their uninfected counterparts, suggesting that the relationship between malaria and certain blood cell indices may be more complex than previously thought [4].

Furthermore, hypoglycaemia is another important complication of severe *P. falciparum* malaria, particularly in children and pregnant women. In sub-Saharan African children where Cameroon is inclusive, 10-20% of children affected with malaria are victimised [12,13]. However, in another study conducted by Ezeugwunne *et al.* [14], there was no significant difference in the mean values of fasting blood glucose levels observed between students with heavy malaria parasitaemia and those with moderate malaria infection. The severity of these biological changes induced by the malaria parasites relies not only on the ability of the parasite to invade and grow in different cells but also on the number of parasites present in the host (i.e., para-sitaemia) [7,8]. The method used for quantifying parasitaemia is simply a count of the number of blood-stage parasites using an optical microscope, on a thick blood film [15].

An exclusive investigation by Ngum Fru *et al.* [16] revealed that about 53% of deaths among children below the age of five are due to malaria-related effects on their biological and haematological parameters. Therefore, impact of malaria parasitaemia on biological parameters remains a significant concern that requires further investigation and understanding.

Malaria is still a major call for concern in Cameroon with the disease being present in all regions of the country [3]. Recently, the Ministry of Public Health has kicked off the malaria vaccine roll out across 42 districts in the country’s 10 regions in public and private health facilities. This vaccine will serve as an additional tool for malaria control as it will specifically target all children aged 6 months and over [17].

This study was designed to determine the prevalence and intensity of malaria infection, and to assess how different biological parameters such as RBC count, haemoglobin concentration, haemat-ocrit, white blood cell count, platelet count and blood sugar level vary with respect to different levels of parasitaemia (low, moderate and high) among children less than 16 years old attending the Nkwen district hospital in northwest Cameroon. This knowledge is crucial for preliminary diagnosis and presumptive treatment [8]. It would also serve as an indicator to monitor the severity of infection thereby helping to guide Government’s efforts in the strategic planning and design of malaria control programmes.

## Materials and Methods

### Ethical considerations

An administrative authorisation to carry out the study was obtained from the North West Regional Delegation of Public Health. Ethical clearance was obtained from the Institutional Review Board of the Faculty of Health Sciences, The University of Bamenda. These documents were presented to the general supervisor of the Nkwen District Hospital where authorisation to carry out the research in their health facility was granted. Guardians and parents of participants were adequately informed of the objectives and benefits of the study and interested persons signed assent forms. Data from participants was used solely for the research, and all records were kept strictly confidential.

### Study area

Nkwen, a locality in the northwest Region of Cameroon with a population of about 40,000 is located some 278 km northwest of Yaounde, the country's capital city. According to Antonio-Nkondjio *et al.* [18], Nkwen, just like other localities in Cameroon, is endemic to malaria with the latest prevalence being 26.7% [18]. Nkwen has higher average environmental temperatures (21.1–22 °C) compared to neighbouring localities like Bambili (16.1–20.0 °C) and Santa (17.1–20.0 °C). It is located at an altitude of 1201–1400 m above sea level and specifically on a latitude of 100 10′0’’ E and longitude of 50 56′0’’ N [19]. This cosmopolitan community is socially stratified with occupants ranging from low to high socioeconomic status. Their main occupation is farming and business.

### Study design and period

The study was a hospital-based cross-sectional study recruiting children who came to consult at the Nkwen district hospital. The study ran for 3 months; from March to May 2023, a period which coincided with the first peak of annual malaria infection.

The study sample size was determined using the Cochran formula [19, 21]:

$$n=\frac{Z^{2}p(1-p)}{e^{2}}$$

Where n = the sample size required; Z = 1.96 conf-dence limit at a desired level of significance (for a 95% confidence interval, CI); p = prevalence of malaria in Bamenda = 26.7% [19]; e = error rate = 0.05.

$$n=\frac{{\left(1.96\right)}^{2}X0.267(1-0.267)}{0.05^{2}}=300.72$$

The minimum sample size estimated was n = 301. Convenient and consecutive sampling was used to select participants after they consented. Excluded from the study were children with a history of antimalarial treatment or other recent treatments for febrile illnesses during the previous two weeks in order to limit bias from false negatives and poor resolution of biological alterations. Also, patients identified with any other infection that may cause anaemia were excluded from the study.

### Data collection

Structured, close-ended questionnaires written in English were administered to obtain demographic information (sex, age, residence) of the participants. Socioeconomic status-related variables (number of house occupants, occupation, and level of education) and knowledge of malaria (signs/symptoms, transmission and prevention) featured in the questionnaire. For those who were not able to read and understand the English language, the questions were orally translated into Pidgin English, the lingua franca of the region.

At enrolment, patients’ facial skin temperatures were measured using an infrared forehead digital thermometer (HTD8813C, Hetaida Technology Co. Ltd, China). The results were immediately recorded on the data sheet in degrees Celsius to the nearest 0.1°C and a participant was considered febrile when he/she had a body temperature ≥37.5°C [19].

Venous blood was collected following the principles of blood collection and used for the research. Approximately 2 mL of blood was collected by venepuncture into a 5 mL sterile disposable syringe and a small drop of the collected blood was placed on an RDT cassette (Abbott Bioline^TM^ Malaria Ag P.f/Pan). The remaining blood in the syringe was dispensed into a labelled ethylene-diamine-tetra-acetate (EDTA) tube for a full blood count (FBC) [19].

The value of blood sugar was measured using a glucometer (On Call Plus Blood Glucose Meter ACON Laboratories, Inc., USA). In this process, a small quantity of the collected blood was immediately dropped on a test strip inserted in a glucometer and the result displayed on the glucometer screen was recorded on the data sheet. Values below 70mg/dL were considered hypoglycaemic [12,13].

The small drop of blood placed on an RDT cassette (Abbott Bioline^TM^ malaria Ag P.f/Pan) was chased to migrate by the addition of diluent buffer and the results were read after 15 minutes. The appearance of two lines (the control and the test line) indicated a positive result. If only the control line appeared, then the test was negative and if only the test line appeared or no lines appear at all, then the test was considered invalid.

In this process, a drop of blood was placed on a labelled grease-free glass slide and using the edge of another glass slide, a circular coin-shaped thick film was made by smearing the drop of blood over the slide in order to rupture all red blood cells and dislodge any parasite present. The slide was then air dried over a light source after which it was stained with 10% Giemsa. The slide was then observed under the oil immersion (x100) objective of a microscope following standard operating procedures. A slide was considered negative after scanning through at least 100 high-power fields without finding a malaria parasite [20]. As a quality control measure, slides were read by two independent par-asitologists in the Nkwen District Hospital, and in case of any disparity they were read by a third party. To quantify parasitaemia, malaria parasites were counted against 200 white blood cells (WBCs) and multiplied by the patient’s actual WBC count as obtained from the FBC. Parasite density per μL of blood was calculated using the formula:

$$\frac{N\text{​}of\text{​}parasites\text{​}counted}{200WBC}X\text{​}Patient's\text{​}WBC\text{​}count$$

Parasitaemia was categorised as low (<1000 para-sites/μL), moderate (1000–4999 parasites/μL) or high (≥5000parasites/μL) [19,21].

A complete blood count was performed on the blood samples of both malaria-positive and negative participants in order to determine haemoglobin concentration, haematocrit, total white blood cells and platelet counts. This was done using a haematological analyser machine (URIT3300, Urit medical Electronic Company Ltd., China), following the manufacturer’s instructions. In this process, the blood samples dispensed in EDTA tubes were mixed by rocking gently on a multitube rotator for about 2 minutes. Meanwhile, the patient’s information (code, name, age and sex) were entered in the machine. Thereafter, blood was aspirated into the machine and the results were immediately displayed and then printed out.

Hb values between 11.0 and 15.0 g/dL were considered normal while values below 11g/dL were tagged anaemic. Anaemia was classified as: mild (Hb 10.9 -10.0 g/dL), moderate (Hb 9.9 – 7.0 g/ dL) and severe (Hb<7.0 g/dL). The normal haematocrit (HCT) range for children is 36-48% [22]. Participants with HCT values <36% were considered anaemic. Anaemia was further categorised as: mild (HCT 36–33%), moderate (HCT 33–30%) and severe (HCT<30%), [23].

The normal WBC value was 5-12 x 10^3^ cells/μL of blood. Total WBC counts less than or greater than the normal range were diagnosed for leukopenia and leucocytosis respectively. For WBC differentials, the normal ranges for lymphocytes, monocytes and granulocytes were (1.0-4.1, 0.1-1.8 and 2.0-7.8) x 10^3^ cells/μL of blood, respectively.

The normal platelet count in children was considered to be 150-300 x 10^3^ cells/μL. Thrombocytopenia was defined as a total platelet count <150 x 10^3^ cells/μL and was further classified as: mild (101-140 x 10^3^ cells/μL), moderate (50-100 x 10^3^ cells/μL) and severe (<50 x 10^3^ cells/μL) [24].

Finally, high-grade fever was recorded for temperatures of 40.1-41.0 °C and hyperpyrexia for temperatures >41.1C °.

### Statistical analysis

Information extracted from the questionnaire and data obtained from the laboratory were entered and analysed using SPSS version 23 (IBM SPSS Inc. Chicago, IL, USA). Data normality was checked using the Shapiro-Wilk normality test. The Chi-square test was used to test the proportion of infected persons (prevalence) with respect to sex and age group. Malaria parasite densities were log-transformed in base 10 to obtain geometric mean parasite density (GMPD) and ANOVA was used to compare the GMPD between age groups (≤5 years, 6-10 years and 11-15 years). The independent sample Student t-test was used to test differences between means of these biological parameters in the two categories (positive and negative). ANOVA was again used to examine the variation of the mean of each biological parameter across the three groups (low, moderate and high parasitaemia). All analyses were done at a 95% Confidence level with p-values <0.05 considered statistically significant.

## Results

Socio-demographic and clinical characteristics of the study participants (Table 1) revealed that, out of the 321 participants who enrolled for the study, 58.3%(187/321) were males while 41.7%(134/321) were females. Most of these, 66.0 % (212/321) were less than or equal to 5 years of age while only 21.8% (70/321) and 12.1% (39/321) were within the age ranges of 6-10 and 11-15 years old, respectively. At enrolment, 58.3 %(187/321) of patients across all age groups were febrile (Table 1). The overall mean temperature was 38.0±1.1 ºC.

**Table 1.** Malaria parasite prevalence and geometric mean parasite density with respect to sex, age and fever status.

Parameter	Number examined (n=321)	Number infected (n=73)	Chi-square test	GMPD±SD/μL of blood*	ANOVA test
**Sex**					
Male	187 (58.3%)	38 (20.3%)	X^2^=1.494 p=0.22	2708.8 ± 5.9	F=1.265 p=0.26
Female	134 (41.7%)	35 (26.1%)		4252.1 ± 6.9	
**Age group (years)**					
≤ 5	212 (66.0%)	39 (18.4%)	X^2^=7.474 p=0.02	3788.8 ± 4.8	F = 4.532 p=0.01
6-10	70 (21.8%)	20 (28.6%)		5377.7 ± 3.2	
11-15	39 (12.1%)	14 (26.9%)		1248.8 ± 17.5	
**Fever status**					
Febrile	187 (58.3%)	69 (36.8%)	X^2^=51.100 p<0.001	6046.4 ± 5.1	F=46.788 p<0.001
Afebrile	134 (41.7%)	4 (2.9%)		3243.4 ± 6.5	

* GMPD = geometric mean parasite density

Out of the 321 participants recruited for the study, 73 tested positive for malaria (all *P. falciparum*) at varying severities giving an overall prevalence of 22.7% (73/321) (Table 1). The prevalence of malaria infection varied with respect to age, sex and body temperature. A higher but not significant (X^2^=1.49, p=0.22) prevalence of 26.1% (35/134) was recorded in females than in males (20.3% (38/187)).

Age-related prevalence was 18.4% (39/212) in children ≤5 years old, 28.6% (20/70) in children aged 610 years and 26.9% (14/39) in children aged 11-15 years old. Statistical analysis showed a significant difference (X^2^=7.47, p=0.02) in the distribution of malaria among the three age groups. Furthermore, out of the 134 participants that were afebrile, only 2.9% (4/134) tested positive for malaria while out of 187 febrile subjects, 36.8% (69/187) had malaria; this difference was significant (X^2^= 51.1, p<0.001).

The overall geometric mean parasite density (GMPD±SD) was 2,670.8±179.9/μL with variations in distribution observed with respect to sex, age and fever prevalence (Table 1). In relation to sex, there was no significant difference (p=0.262) in the GMPD±SD even though females had a higher GMPD±SD (4,252.1 ± 6.9/μL) than males (2,708.8 ± 5.9/μL). With respect to age, the GMPD(±SD) was highest (5,377.7 ± 3.2/μL) in children aged 6-11 years. It was moderate in children ≤ 5 years old (3788.7 ± 4.8/μL) and lowest (1,248.8 ± 17.6/μL) in children aged 11-15 years old. These differences were statistically significant (P=0.011). The GMPD±SD was also significantly higher in febrile children (6,046.4 ± 5.1/μL) than in afebrile children (3,243.4 ± 6.5/μL)(p<0.001).

Overall, children who were positive for malaria showed a significant (p<0.05) reduction in values of most of their biological parameters when compared with their negative counterparts. For RBC count, Hb concentration and HCT, the statistical differences were (t=12.06; p<0.001), (t=8.23; p<0.001) and (t=3.69; p<0.001) (Table 2). The mean blood sugar level of malaria-positive children (90.29 ±21.60 mg/ dL) was equally lower than that for negative children (101.67 ±19.33 mg/dL) even though it did not go below the cut-off value of 70 mg/dL to be qualified as hypoglycaemia. As shown in Table 2, this difference was not statistically significant (t=4.05; p=0.32). In addition, the results showed a signif-cant reduction in means of the total WBCs count between positive and negative children (t=3.25; p=0.01) but did not go below this cut-off value for it to be qualified as leucopoenia. Similarly, different leukocyte components were also significantly affected. The mean granulocyte, lymphocyte, and monocyte counts were all decreased in malaria-positive children when compared with their negative counterparts. Apart from the mean granulocyte count whose difference was not statistically signifcant (t=0.37; p=0.71), lymphocytes and monocytes recorded statistical differences in their mean values (p<0.05) between positive and negative children (Table 2). The mean platelet count of malaria-positive children (134.63±101.06x109/L) was lower than that of negative children (348.97±131.06x109/L). This difference was statistically significant (t=12.88; p<0.001) (Table 2).

**Table 2.** Comparison of mean biological parameters in malaria-positive (n=73) and negative children (n=248).

Parameter	Status	Mean (SD)	t-test / p-value
RBC count x 10^12^/L	Positive	3.91 (0.33)	12.06^a^ / <0.001
	Negative	4.59 (0.38)	
Hb concentration (g/dL)	Positive	10.15 (1.42)	8.23^a^ / <0.001
	Negative	11.65 (1.16)	
Haematocrit (%)	Positive	34.23 (4.62)	3.69^a^ / <0.001
	Negative	36.45 (4.08)	
Glycaemia (mg/dL)	Positive	90.29 (21.60)	4.05^a^ / 0.32
	Negative	101.67 (19.33)	
tWBC count x 10^9^/L	Positive	6.69 (3.14)	3.25^a^ / 0.01
	Negative	8.09 (3.53)	
Lymphocyte count x 10^9^/L	Positive	1.07 (0.90)	7.46^b^ / <0.001
	Negative	2.49 (1.55)	
Mid-range x 10^9^/L	Positive	0.49 (0.32)	3.69^b^ / <0.001
	Negative	0.73 (0.48)	
Granulocytes x 10^9^/L	Positive	4.88 (2.60)	0.37^a^ / 0.71
	Negative	5.01 (2.85)	
Platelet count x 10^9^/L	Positive	134.63 (101.1)	12.88^b^ / <0.001
	Negative	348.97 (131.1)	

RBC: Red blood cell; Hb = Haemoglobin; tWBC = total white blood cell count, ^a^ t-test values for unequal variances (from Levene's Test for Equality of Variances); ^b^ t-test values for equal variances (from Levene's Test for Equality of Variances)

There was a strong impact of different levels of parasitaemia on the means of various biological parameters, albeit to different extents (Table 3). There was a significant difference (F=43.56, p<0.001) in the mean red blood cell count among the three groups of infected participants (low, moderate and high parasitaemia).

**Table 3.** Variation of biological parameters (Mean (SD)) with respect to parasitaemia (low (n=18), moderate (n=25) and high (n=30).

MPD category	RBC x 10^12^/L	Hb (g/dL)	HCT(%)	Gly-caemia (mg/dL)	tWBC x 10^9^/L	Lym x 10^9^/L	Mid-range x 10^9^/L	Gran x 10^9^/L	Platelets x 10^9^/L
Low*	3.9	10.3	34.7	88.2	5.4	1.1	0.5	3.7	207.2
	(0.32)	(0.6)	(5.2)	(21.1)	(0.9)	(0.9)	(0.3)	(1.2)	(111.3)
Moderate	3.8	10.2	33.9	91.6	6.6	0.8	0.4	4.8	133.4
	(0.34)	(1.0)	(4.6)	(25.7)	(2.9)	(0.4)	(0.2)	(2.4)	(91.2)
High	3.7	9.9	34.2	90.5	6.9	1.2	0.5	5.2	76.1
	(0.29)	(1.9)	(4.5)	(18.6)	(3.3)	(1.1)	(0.3)	(2.6)	(32.6)
F-value	43.56	32.06	5.08	6.01	5.25	19.51	6.05	1.50	65.65
p	<0.001	<0.001	0.02	0.01	0.02	<0.001	0.01	0.214	<0.001
Linearity (L)	<0.001	<0.001	0.067	0.082	<0.001	0.062	0.834	<0.001	<0.001

MPD = malaria parasite density; RBC = red blood cells; Hb = haemoglobin; HCT = haematocrit; Lym = lymphocytes; tWBC = total white blood cell count; Gran = granulocytes. * Low = <1000 parasites/μL), moderate = 1000–4999 para-sites/μL), high = ≥5000parasites/μL).

Children with low parasitaemia had their mean platelet counts (207.22 ±111.27 x109/L) within the normal range (150-300 x109/L) whereas those with moderate and high parasitaemia suffered from mild (101-449 x109/L) and moderate (50-100 x109/ L) thrombocytopenia respectively.

At low parasitaemia, the average RBC count drops from normal to 3.9828 ±.31769 x 1012/L (Table 3). The value continued to drop with increasing levels of parasitaemia in a perfectly significant linear manner (L=0.000). The mean Hb concentration showed a progressive and a perfectly linear (L= 0.00) drop with increasing levels of parasitaemia. As shown in Table 3, children with low and moderate parasitaemia had mild anaemia (10.0 -10.9 g/ dL) while those with high parasitaemia had moderate anaemia (7.0-9.9 g/dL). The results equally showed a variation in the mean blood sugar levels among the three groups of infected participants. This reduction showed a significant difference between different levels of parasitaemia though it did not go below the normal blood sugar range (70-140 mg/dL) for it to be tagged as hypoglycaemia. Therefore, a reduction in blood sugar levels was evident in malaria-positive children but hypoglycaemia was not noticed. Unlike haemoglobin, haematocrit and glycaemia, the tWBC showed a perfectly linear (L=0.00) direct relationship with parasitaemia tWBC was opposite. Among the positive children, the mean tWBC was lowest in children diagnosed with low parasitaemia and gradually increased as parasitaemia increased. For differential WBC count, lymphocytes and monocytes were high at low parasitaemia, low at moderate parasitaemia and again high at high parasitaemia. Granulocytes, just like tWBC, showed a direct linear relationship with increasing levels of parasitaemia.

## Discussion

The overall malaria prevalence of 22.7% reveals that malaria continues to be a major public health problem in Nkwen area accounting for high morbidity and mortality rates in spite of increased efforts directed towards control measures. This prevalence was similar to a 19.2% prevalence obtained in a neighbouring locality (Bambili) by Payne *et al.* [25] in their quest to establish the prevalence of malaria among school children. The 22.7% obtained in this study remains lower than the national prevalence (30.3%) in Cameroon [3]. The lower prevalence observed in our study may be due to the intensive nationwide free distribution of insecticide-treated bed nets (ITNs). A decline in malaria burden attributed to the use of interventions such as ITNs and LLINs has also been reported in some malaria-endemic countries such as Kenya and Tanzania [8,25]. The decline in malaria parasite prevalence may also be credited to a significant switch in the treatment of malaria from sul-phadoxine-pyrimethamine due to widespread resistance to more effective artemisinin-based combination therapies as first-line treatment since 2004 [27].

Prevalence varied with respect to gender. The prevalence in females (26.6%) was slightly higher than that in their male counterparts (20.3%) although the difference was not statistically signif-cant. These results contrast with those obtained by Ngum *et al.* [19] where males recorded an insignif-cant higher prevalence of malaria (38.6%) than females (32.1%).

Statistical analysis also showed a significant difference in the distribution of malaria among the three age groups, being highest in children aged 6-10 years old. This finding was in line with those of Teh *et al.* [5] and Ngum *et al.* [19], who noticed that the prevalence of falciparum malaria was highest among a similar age group when compared with the under-five and 10–14 years age group. The significant increase in prevalence suggests that children in this age range (6-10 years) are more exposed given that at this age they start spending more time outdoors during evenings. Maternal care over these children reduces as mothers now direct more attention toward younger children. It is also probable that these children experienced less repeated exposure to malaria. Malaria is a disease in which immunity is not sterile and the level of immunity a person shows depends on the number of episodes of clinical malaria that an individual has experienced [28].

Fever is highly sensitive but a poorly specific indicator of *P. falciparum* infections. In our study, 94.5% of malaria-positive children were febrile confirming a strong implication of fever in malaria infections. A similar observation was reported by Kwenti *et al.* [29] in a study on children of similar age groups. The fever and chills associated with malaria infection are attributed to the rupture of erythrocytic-stage schizonts [30]. Recently, pundits have focused on the insoluble product of haemoglobin digestion (hemozoin), which is produced at high concentrations during the intra-erythrocytic stage of the malaria life cycle and is released during erythrocyte rupture. On the other hand, a good number of febrile subjects (63.1%) were malaria parasite negative. The high temperatures in these negative children could be attributed to other infections, such as co-infections with other parasites, viruses or bacteria, which were beyond the scope of this study.

The overall GMPD was 2,670.85/μL of blood. This value was higher as compared to that obtained earlier in children of a similar age group residing at different altitudes along the slope of Mount Cameroon [5]. The high GMPD obtained in our study could be attributed to the fact that our study was conducted between March and May; a period which coincided with the first peak of annual malaria transmission. Further analysis with respect to sex and age revealed that female children had a higher GMPD (4,252.1/μL) than male children (2,708.8/μL) even though the difference was not statistically significant. It was equally noticed that children aged 6-10 years old were most affected with 50% having high parasite densities while 45% had moderate parasite densities. These statistics were significantly higher than for children ≤ 5 years and 11-15 years. This could probably be due to intensive malaria control including free ITNs and ACTs for the under-five age group in all government health centres in the country. This might have decreased malaria exposure in under-fives, thereby delaying the development of malaria protective immunity. The age-related decrease in malaria parasite density observed as children enter the age range of 11-15 years is probably related to the acquisition of protective immunity due to repeated infections over time. These results are consistent with those of Kimbi *et al.* [8] who found that a majority (184/246) of their participants diagnosed with high malaria parasitaemia were >7 years of age.

The presence of malaria parasites was found to be associated with lower RBC, Hb, Hct, blood sugar, WBC and platelet counts. Generally, children who tested positive for *P. falciparum* in the entire study registered lower RBC indices (RBC, Hb, and Hct) when compared with their negative counterparts. Even though *Plasmodium* infection is thought to lower erythrocyte counts by inducing haemolysis, increased clearance of both parasitised and non-infected erythrocytes in the spleen during malaria infection might also be a contributing factor. Further exploration revealed that the means of RBC indices reduces linearly as parasite density increases. This is probably because an increase in parasite density leads to a more intense attack and destruction of more RBCs. Similar observations were made earlier by Kimbi et al. [8], who noticed an inverse effect of the parasite density on the mean RBC count. In similar studies [5,6,14,21] there was a significant decrease in the mean haemoglobin level in malaria-infected children compared to negative controls.

Hypoglycaemia is generally believed to be a complication associated with severe malaria. This study revealed a significant reduction in the mean blood sugar level of malaria-positive children when compared with their negative counterparts although the reduction did not go below the cut-off value for it to be qualified as hypoglycaemia. In a study conducted by Adamu *et al.* [31], the reduction in serum glucose observed was attributed to the parasite’s utilisation of glucose. This hypothesis agrees with the fact that *Plasmodium* does not store energy in the form of glycogen and relies on an exogenous glucose supply [32]. Secondly, erythrocytes exhibit a substantial increase in membrane permeability to low molecular weight sugar in a bid to cope with the stress posed by the parasites leading to hypoglycaemia [32]. This reduction, however, did not show any significance variation with different levels of parasitaemia.

Consistent with other investigations [4,19,33] an increase in WBC counts was found to be associated with malaria infection. Among the positive children, a direct linear relationship was observed between tWBC count and parasite density. This was consistent with previous studies [34,35] that reported a consistent positive relationship between WBC count and parasite density. As parasitaemia increases, the immune system intensifies the product ion of WBCs , e spe c i a ll y gr anul o c y t e s (eosinophils), leading to the elevated leukocyte counts. The decrease in tWBC count witnessed in this study could be explained by the redistribution of these cells to infected sites such as the liver or their destruction due to Fas-induced apoptosis during malaria infection.

Findings from this study also revealed that the mean platelet count of malaria-positive children was significantly lower than that of negative children. Similar to RBC count and Hb concentration, a significant inverse linear relationship was observed in the mean platelet count among patients with different levels of parasitaemia. These results were consistent with those of Muhammad *et al.* [36] and Angesom *et al.* [37] who stated that thrombocytopenia is the most common haematological abnormality in patients with acute malaria. The pathogenesis of thrombocytopenia is thought to involve a plethora of processes, some of which include splenic pooling and sequestration of platelets, parasite invasion and phagocytosis of platelets, and oxidative stress [38].

## Conclusions

Malaria continues to be a major public health problem in the Nkwen locality with variations in prevalence distribution observed with respect to sex, age and fever. Malaria-positive children were found to have lower RBC count, Hb, Hct, blood sugar, and platelet counts when compared with their negative counterparts. Among the positive children, RBC count, Hct, Hb, lymphocyte and platelet count each showed a significant linear decrease with increasing levels of parasitaemia while tWBC and granu-locyte count each showed a reverse. Changes in these biological parameters during malaria infection are sensitive but poorly specific indicators of malaria infection since they may overlap with other infections caused by other parasites, viruses or bacteria. Children 6-10 years of age deserve particular attention when planning malaria interventions.
